# Patient-reported outcomes and skeletal symptoms in multiple myeloma stratified by proteasome inhibitor-based therapy: a prospective interdisciplinary cohort study

**DOI:** 10.1186/s12885-026-17177-7

**Published:** 2026-10-15

**Authors:** Julian P. Maier, Carlotta Pietsch, Hagen Schmal, Ioannis Ntanasis-Stathopoulos, Evangelos Terpos, Gabriele Ihorst, Ralph Wäsch, Monika Engelhardt, Georg W. Herget

**Affiliations:** 1 Department of Orthopedics and Trauma Surgery, Faculty of Medicine, Medical Center - University of Freiburg, Freiburg, Germany; 2 Comprehensive Cancer Center Freiburg (CCCF), Faculty of Medicine, Medical Center - University of Freiburg, Freiburg, Germany; 3 Department of Clinical Therapeutics, School of Medicine, National and Kapodistrian University of Athens, Athens, Greece; 4 Clinical Trials Unit, Medical Center, Faculty of Medicine, Medical Clinic, University of Freiburg, Freiburg, Germany; 5 Department of Medicine, Hematology and Oncology, Faculty of Medicine, Medical Center - University of Freiburg, Freiburg, Germany

**Keywords:** Multiple myeloma, Quality of life, Bone pain, Osteolysis, Proteasome inhibitor, Antiresorptive therapy.

## Abstract

**Background:**

Multiple myeloma (MM) frequently causes osteolytic bone disease and skeletal symptoms. Proteasome inhibitors (PIs) are central to MM treatment and in routine practice, PI-based therapy is preferentially selected for patients with more active or symptomatic disease. Hereby, PIs may also exert bone-modulating effects beyond their antimyeloma activity. Whether these biological effects are reflected in patient-reported outcomes or skeletal symptoms during routine care remains unclear.

**Methods:**

We prospectively enrolled 209 patients with MM during routine interdisciplinary follow-up and stratified them by current PI-based versus non-PI-based treatment status. Sixty-nine patients were reassessed after at least 6 months. Patient-reported outcomes and skeletal symptoms were evaluated using multivariable regression and sensitivity analyses addressing baseline imbalances and treatment selection.

**Results:**

Seventy-eight patients (37%) were currently receiving PI-based treatment. In adjusted analyses, current PI-based treatment status was not associated with Quality of Life (β = -0.08, 95% CI -0.45 to 0.29; *p* = 0.662), health-related status (β = -0.06, 95% CI -0.42 to 0.30; *p* = 0.740), bone pain (OR 0.92, 95% CI 0.48 to 1.74; *p* = 0.791), or prevalent osteolytic lesions (OR 1.26, 95% CI 0.53 to 2.96; *p* = 0.601). QoL and health-related status remained comparable during follow-up. MM-related bone pain was numerically more frequent among patients currently receiving PI-based treatment (46% vs. 33%), although the association did not reach statistical significance after multivariable adjustment (OR 1.80, 95% CI 0.93 to 3.48; *p* = 0.081).

**Conclusions:**

In this prospective real-world cohort, current PI-based treatment status was not associated with measurable differences in global patient-reported outcomes, overall bone pain, or prevalent osteolytic lesions compared with non-PI-based treatment. MM-related bone pain was numerically more frequent among patients currently receiving PI-based treatment, although the association did not reach statistical significance after multivariable adjustment. These findings provide a real-world description of patient-reported and skeletal outcomes according to current treatment status but do not establish whether the biological effects of proteasome inhibitors on bone remodeling translate into clinically meaningful patient-reported or structural skeletal benefits.

**Supplementary Information:**

The online version contains supplementary material available at https://doi.org/10.1186/s12885-026-17177-7.

## Introduction

Multiple myeloma (MM), the second most common malignant hematologic neoplasm, is characterized by the clonal proliferation of malignant plasma cells within the bone marrow [1, 2]. These cells profoundly alter the bone marrow microenvironment and disrupt the balance between osteoclast-mediated bone resorption and osteoblast-driven bone formation through complex cellular and molecular mechanisms [1, 3]. Increased osteoclast activity combined with suppressed osteoblast differentiation result in progressive bone destruction and constitutes a defining feature of MM-associated bone disease [3, 4].

Clinically, altered bone remodeling leads to osteolytic lesions, which are present in approximately 80% of patients at initial diagnosis and in up to 90% over the disease course [2, 5–8]. MM-related bone disease is associated with significant morbidity, including bone pain, impaired mobility, reduced quality of life (QoL), and a high risk of skeletal-related events (SREs) such as pathological fractures, spinal cord compression, and the need for radiotherapy or surgical intervention [9, 10]. Among these complications, bone pain represents one of the most debilitating and clinically relevant symptoms for patients with MM [11].

Proteasome inhibitors (PI), such as bortezomib, carfilzomib, and ixazomib, have become a cornerstone of MM therapy and may exert effects beyond tumor cell control by directly modulating bone remodeling [12]. In contrast to antiresorptive therapies such as bisphosphonates and the RANKL inhibitor denosumab, proteasome inhibition has been shown to suppress osteoclast differentiation and function while promoting osteoblast activity and bone formation [13–15]. Preclinical and translational studies indicate that PI treatment can partially restore bone homeostasis and improve structural bone parameters in MM [12, 16–18]. Further clinical studies have shown that PI-based regimens may improve biochemical markers of bone remodeling compared to non-PI-based therapy [19]. However, whether these bone-modulating effects translate into measurable clinical benefits, such as improved patient-reported outcomes, reduced bone pain, or a lower osteolytic lesion burden, remains unclear. This question is particularly relevant in routine follow-up care, where PI-based regimens are administered across different treatment phases in clinically heterogeneous patient populations. In fact, isolating treatment-specific effects on bone pain in MM is methodologically challenging because pain is inherently multifactorial and influenced by disease activity, skeletal involvement, prior treatment, and other patient-specific factors. Distinguishing bone pain attributed to MM-related skeletal involvement from pain of other origin was therefore considered clinically relevant when examining skeletal symptoms according to current PI treatment status. Against this background, our study aimed to characterize patient-reported outcome measures and the descriptive evaluation of skeletal symptoms, including bone pain and osteolytic lesions, in patients with MM receiving routine follow-up care, stratified according to current PI-based versus non-PI-based treatment regimens.

## Materials and methods

### Study design and patient selection

This observational cohort study included 209 patients with MM, consecutively enrolled between 02/09/2022 and 06/21/2024 in accordance with the Declaration of Helsinki Principles and Good Clinical Practice. All patients gave their written informed consent for institutional-initiated research studies and analyses of clinical outcome studies, conforming to our institutional review board guidelines (amendment 11/2025, Ethical committee protocol No. 30/18).

**Fig. 1:**
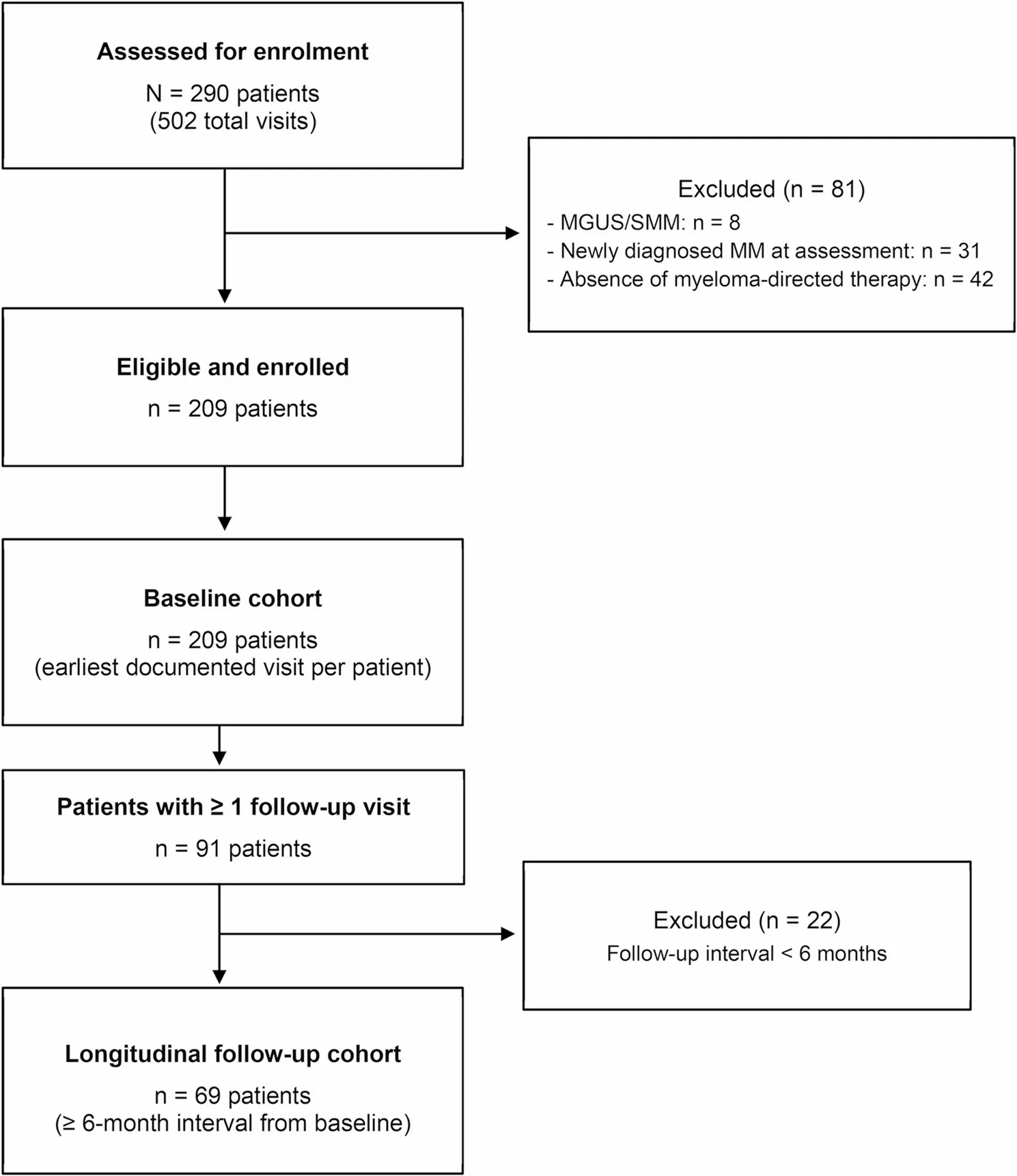
Flow-Chart of patient screening, study enrollment, and longitudinal follow-up. Of 290 patients assessed for enrollment, 209 were eligible and included in the baseline cohort. Ninety-one patients had at least one follow-up visit, of whom 69 met the prespecified minimum follow-up interval of 6 months and were included in the longitudinal cohort. Reasons for exclusion are shown in the flowchart. MGUS, monoclonal gammopathy of undetermined significance; SMM, smouldering multiple myeloma

Eligible participants were patients with a confirmed diagnosis of MM. Exclusion criteria included newly diagnosed MM at presentation, monoclonal gammopathy of undetermined significance (MGUS), smouldering multiple myeloma (SMM), absence of myeloma-directed therapy, or any severe medical or psychiatric condition impairing consciousness or study participation (Fig. 1). Patients whose most recent myeloma-directed treatment consisted of high-dose therapy followed by ASCT were considered eligible. No a priori sample-size calculation was performed. Study size was determined by the number of eligible patients enrolled during the predefined recruitment period. For the prospective interview-based component, a structured questionnaire developed and previously published by our interdisciplinary multiple myeloma research group [11], was administered to patients during routine outpatient follow-up visits.

For follow-up analyses, repeated patient assessments were included only if the interval between visits was at least 6 months. This minimum interval was selected as a pragmatic threshold to provide sufficient temporal separation between assessments and reduce the influence of short-term fluctuations in symptoms and patient-reported status. In patients with multiple eligible reassessments, only the latest available follow-up assessment was included to maximize follow-up duration and avoid multiple observations per individual. Assessments less than 6 months after baseline were excluded. Time intervals were calculated in months based on the documented assessment dates.

The questionnaire included demographics (age at initial diagnosis, sex), disease characteristics: CRAB symptoms (hypercalcemia, renal failure, anemia, bone lesions), International Staging System (ISS) stage, duration of disease, osteolysis, prior anti-myeloma therapy, pre-existing orthopedic diagnosis, occurrence of skeletal pain, and patient-reported outcomes (PROMs), including QoL and health-related status. QoL and health-related status were assessed using single-item 7-point Likert scales (range 1–7), with higher values indicating better perceived status [11]. These pragmatic global ratings were derived from our previously published interdisciplinary questionnaire [11] and were designed for brief and practical administration during routine outpatient follow-up. Their advantages include ease of use, minimal respondent burden, and straightforward longitudinal comparison. ISS was used as the disease-stage variable, as it was prospectively and consistently available across the cohort, allowing uniform adjustment in multivariable analyses.

All patients were reviewed within an interdisciplinary tumor board as part of routine clinical care. MM-related bone pain was defined by concordance between pain location and osteolytic involvement on available imaging, together with the formal radiological report. Among 202 assessments with imaging suitable for skeletal evaluation, whole-body low-dose CT predominated (*n* = 183, 90.6%), followed by PET/PET-CT (*n* = 9, 4.5%), MRI (*n* = 7, 3.5%), and conventional radiography (*n* = 3, 1.5%). MM-related bone pain was analyzed primarily in the entire cohort, with patients without bone pain classified as not having MM-related bone pain, and secondarily among patients reporting bone pain, to assess whether an existing pain complaint was attributed to MM rather than to other causes.

Osteolytic lesions were classified as present if documented on assessable imaging, irrespective of radiological stability. Because this binary endpoint reflected prevalent structural disease rather than temporal change, new or progressive lesions were assessed separately within the progressive-disease variable.

Progressive disease was adjudicated hierarchically. When current imaging obtained within 3 months was available and suitable for comparison, progression was defined by new and/or radiologically progressing osteolytic lesions. Otherwise, adjudication relied on documented clinical evidence suggestive of progression, including abnormal serology, worsening renal function, new or worsening skeletal pain, or systemic symptoms. In the absence of radiological or clinical evidence of progression, progressive disease was classified as absent. No formal IMWG imaging-response or relapse criteria were applied.

Clinical and treatment-related data were extracted from electronic medical records documented in our hospital database and pseudonymized prior to analysis. Proteasome inhibitor (PI)-based therapy and antiresorptive therapy (ART) were coded as binary variables (yes/no) according to treatment status at the time of assessment. Exposure thus reflects current treatment status rather than cumulative exposure, and the terms “PI-treated” and “non-PI-treated” refer to this status throughout. Patients’ characteristics and clinical outcome parameters were documented at the time of assessment.

### Statistical analysis

Baseline characteristics were compared between patients receiving PI-based therapy and those not receiving PI-based therapy at study inclusion. Continuous variables are reported as medians with interquartile range (IQR) and were compared using the Mann-Whitney U test. Categorical variables are presented as counts and percentages and were compared using the Yates-corrected Chi-square test. Fisher’s exact test was used if any expected cell count was less than 5. No single confirmatory primary outcome was prespecified. QoL, health-related status, general bone pain, MM-related bone pain, and prevalent osteolytic lesions were considered the principal outcomes of interest. Pain duration and longitudinal changes were considered secondary analyses. PI × ART interaction analyses were exploratory, whereas propensity-score weighting and analyses restricted according to progressive disease status were performed as sensitivity analyses.

Associations between current PI-based treatment status and clinical outcome parameters were assessed using univariate and multivariable regression models. Binary outcomes (general bone pain, MM-related bone pain, and osteolytic lesions) were analyzed using logistic regression, and results are reported as odds ratios (OR) with 95% confidence intervals (CI). Continuous outcomes (pain duration, disease duration, quality of life [QoL], and health-related status [HRS]) were analyzed using linear regression models and are presented as β coefficients (ß) with 95% CI. Pain duration was log-transformed (log1p) to account for right-skewed distribution. Multivariable models were adjusted for age, sex, CRAB symptoms, ISS stage, orthopedic comorbidity, and ASCT as part of primary therapy. For patient-reported outcomes (QoL and HRS), disease duration and ART were additionally included as covariates. To account for combinational treatment regimens, additional interaction analyses between PI and ART were performed by including a multiplicative interaction term (PI x ART).

For longitudinal analyses, patient-related changes between baseline and follow-up were first assessed using McNemar’s exact test for binary outcomes and Wilcoxon signed-rank tests for ordinal or non-normally distributed continuous outcomes. Secondly, adjusted linear regression of the change score on baseline PI and ART therapy was performed for continuous patient-reported outcomes (ΔQoL, ΔHRS), controlling for age, sex, ISS stage, CRAB symptoms, orthopedic comorbidity, disease duration at baseline, and ASCT as part of primary therapy. An analogous interaction model included the interaction (PI x ART) term. For the binary outcomes at follow-up (bone pain, MM-related bone pain, osteolytic lesions), adjusted logistic regression was performed with baseline PI and ART as main exposures and age and sex at baseline as covariates.

To assess robustness to potential confounding by indication, a propensity score-based sensitivity analysis was performed for each primary outcome, estimating the association with current PI-based treatment status. Propensity scores for current PI-based treatment status were estimated using logistic regression including age, sex, CRAB symptoms, ISS stage, orthopedic comorbidity, disease duration, progressive disease status, ASCT as part of primary therapy, and current ART as baseline covariates. Stabilized inverse-probability-of-treatment weights (IPTW) were computed and truncated at the 1st and 99th percentiles to limit the influence of extreme weights. Covariate balance after weighting was evaluated using standardized mean differences, with absolute values < 0.10 considered acceptable. The association of each outcome with current PI treatment status was then re-estimated by weighted regression of the outcome on PI-based treatment status as the sole predictor – weighted logistic regression for binary outcomes and weighted linear regression for continuous outcomes. Robust standard errors (HC3) were applied to all regression models to account for potential heteroscedasticity. Missing outcome or covariate data were not imputed. Each regression model was based on complete cases for the variables included in that model, with the corresponding analytical sample size reported for each analysis.

All analyses and plot visualizations were conducted in R version 4.4 (R Foundation for Statistical Computing, Vienna, Austria) using the packages *dplyr*, *sandwich*, *lmtest*, *ggplot2*, *WeightIt* (inverse-probability-of-treatment weighting), and *cobalt* (covariate balance assessment). Plots were graphically refined using Affinity Publisher (Serif Europe Ltd., Nottingham, UK). A two-sided p-value < 0.05 was considered statistically significant.

## Results

### Baseline characteristics stratified by current treatment regimen

A total of 209 patients were included, of whom 78 (37%) were currently receiving PI-based therapy at study inclusion and 131 (63%) non-PI-based treatment. Median age (68 vs. 67 years; *p* = 0.247) and sex distribution were comparable between groups. Patients with current PI-based treatment status had a shorter median disease duration at study inclusion (20 [IQR 10–43] vs. 34 [IQR 12–83] months; *p* = 0.007). CRAB features and baseline prevalence of osteolytic lesions did not differ between groups (84% vs. 81%; *p* = 0.643). ISS stage III was more frequent among patients with current PI-based treatment status, although the difference was statistically not significant (37% vs. 28%; *p* = 0.074). Progressive disease was present in 34 of 209 (16%) baseline assessments and did not differ significantly between treatment groups (18% vs. 15%; *p* = 0.753). Of these 34 cases, 18 were confirmed radiologically by new or increasing osteolytic lesions on current imaging, 4 reflected partial radiological progress, and 12 were identified from documented clinical diagnostics such as abnormal serology (*n* = 10) or worsening renal function (*n* = 2) together with an accompanying clinical finding like systemic symptoms (*n* = 6), new or worsening skeletal pain (*n* = 3), renal insufficiency (*n* = 2), or increased susceptibility to infection (*n* = 1). ASCT as part of primary therapy was less frequent among patients currently receiving PI-based therapy vs. those not (58% vs. 79%; *p* = 0.002). Concomitant antimyeloma therapies at study inclusion also differed by treatment group: immunomodulatory drug (IMiD) use was less frequent among patients with current PI-based treatment status (6% vs. 60%; *p* < 0.001), whereas immunotherapy (monoclonal or bispecific antibody) use (31% vs. 43%; *p* = 0.115), CAR-T cell therapy (0% vs. 3%; *p* = 0.299), and antiresorptive therapy (49% vs. 56%; *p* = 0.402) did not differ significantly between groups (Suppl. Table 1). Further baseline characteristics are presented in Table 1.

**Table 1:** Patient characteristics in the full cohort (n = 209), stratified by current proteasome inhibitor (PI) therapy and antiresorptive therapy (ART). Continuous variables are presented as median [IQR] and categorical variables as counts n (%). Osteolysis denominators reflect non-missing imaging assessments (7 patients with missing imaging: PI = 1, non-PI = 6). Use of individual proteasome inhibitors as well as concomitant therapies at the time of assessment, including immunomodulatory drugs, immunotherapy, and chimeric antigen receptor T-cell (CAR-T) therapy, were partly imbalanced between groups as expected by treatment-selection patterns and are summarized in Suppl. Tab. 1-3. MM, multiple myeloma; ID, initial diagnosis; ISS, International Staging System; CRAB, hypercalcemia/renal insufficiency/anemia/bone lesions; ASCT, autologous stem cell transplantation. †P-values compare PI vs. non-PI group

	Total (*n* = 209)		PI (*n* = 78)	PI+/ART+ (*n* = 38)	PI+/ART- (*n* = 40)	Non-PI (*n* = 131)	PI-/ART+ (*n* = 73)	PI-/ART- (*n* = 58)	*P* value†
**Median Age**, years [IQR]	67 [60, 73]		**68 [62, 74]**	67 [63, 72]	69 [62, 75]	**67 [60, 73]**	67 [58, 74]	66 [60, 71]	0.247
**Male sex**, n (%)	132 (63%)		**45 (58%)**	21 (55%)	24 (60%)	**87 (66%)**	45 (62%)	42 (72%)	0.265
**Disease duration** (months)	27 [11, 74]		**20 [10**,** 43]**	26 [12, 49]	15 [5, 37]	**34 [12**,** 83]**	42 [20, 79]	20 [7, 86]	**0.007**
**CRAB symptoms at ID**, n									0.584
0	10 (5%)		**2 (3%)**	0 (0%)	2 (5%)	**8 (6%)**	4 (6%)	4 (7%)	
1	68 (33%)		**23 (30%)**	11 (29%)	12 (30%)	**45 (34%)**	30 (41%)	15 (26%)	
2	70 (34%)		**27 (35%)**	12 (33%)	15 (38%)	**43 (33%)**	26 (36%)	17 (29%)	
3	38 (18%)		**15 (19%)**	8 (21%)	7 (18%)	**23 (18%)**	8 (11%)	15 (26%)	
4	23 (11%)		**11 (14%)**	7 (18%)	4 (10%)	**12 (9%)**	5 (7%)	7 (12%)	
**ISS at ID: I / II / III**									0.074
I	71 (34%)		**19 (24%)**	8 (21%)	11 (28%)	**52 (40%)**	36 (49%)	16 (28%)	
II	72 (34%)		**30 (39%)**	14 (37%)	16 (40%)	**42 (32%)**	22 (30%)	20 (35%)	
III	66 (32%)		**29 (37%)**	16 (42%)	13 (33%)	**37 (28%)**	15 (21%)	22 (38%)	
**Osteolytic lesions**, n (%)	166/202 (82%)		**65/77 (84%)**	34/38 (90%)	31/39 (80%)	**101/125 (81%)**	56/69 (81%)	45/56 (80%)	0.643
**Orthopedic comorbidity**, n (%)	100 (48%)		**39 (50%)**	21 (55%)	18 (45%)	**61 (47%)**	34 (47%)	27 (47%)	0.736
**Progressive disease status**, n (%)	34 (16%)		**14 (18%)**	4 (11%)	10 (25%)	**20 (15%)**	11 (15%)	9 (16%)	0.753
**Primary therapy** ASCT	148 (71%)		**45 (58%)**	29 (76%)	16 (40%)	**103 (79%)**	57 (78%)	46 (79%)	**0.002**

### Patient-reported outcomes and skeletal symptoms by current treatment regimen

Patient-reported outcomes and pain characteristics at study inclusion stratified by treatment regimen are summarized in Table 2. Quality of Life (QoL) and health-related status were assessed as PROMs in all patients (*n* = 209). Median QoL was 5 (IQR 4–6) in both treatment groups, while median health-related status was 4 (IQR 4–5) in PI-treated and 5 (IQR 4–5) in non-PI-treated patients. Overall, 109 of 209 patients (52%) reported bone pain. Its prevalence was comparable between groups (54% vs. 51%; *p* = 0.814). In the entire cohort, MM-related bone pain was numerically more frequent among patients currently receiving PI-based therapy (46% vs. 33%; *p* = 0.076). Among the 109 patients reporting bone pain, 79 (73%) had pain attributed to MM based on current imaging findings, with a higher proportion in the PI-based than in the non-PI treatment group (86% vs. 64%; *p* = 0.026). Median pain duration was comparable between groups (5 [IQR 2–11] vs. 3 [IQR 2–6] months; *p* = 0.292).

**Table 2:** Pain characteristics and patient-reported outcomes at study inclusion (*n* = 209), stratified by therapy group. Continuous variables are presented as median [IQR] and categorical variables as counts n (%). *Pain duration assessed only in patients reporting bone pain (*n* = 109). ^#^Percentage calculated within the full cohort (*n* = 209); patients without bone pain are classified as not having MM-related bone pain. ^†^P-values compare PI vs. non-PI group

	Total (*n* = 209)	PI (*n* = 78)	PI+/ART+ (*n* = 38)	PI+/ART- (*n* = 40)	Non-PI (*n* = 131)	PI-/ART+ (*n* = 73)	PI-/ART- (*n* = 58)	*P* value†
**Quality of life** (1–7), median (IQR)	5 [4, 6]	**5 [4, 6]**	5 [4, 6]	4 [3, 6]	**5 [4, 6]**	5 [4, 6]	5 [4, 6]	0.383
**Health-related status** (1–7), median (IQR)	5 [4, 5]	**4 [4**,** 5]**	5 [4, 6]	4 [4, 5]	**5 [4**,** 5]**	5 [4, 6]	5 [4, 5]	0.442
**Bone pain**, n (%)	109 (52%)	**42 (54%)**	19 (50%)	23 (58%)	**67 (51%)**	41 (56%)	26 (45%)	0.814
**MM-related bone pain**, n (%)^#^	79 (38%)	**36 (46%)**	15 (39%)	21 (53%)	**43 (33%)**	26 (36%)	17 (29%)	0.076
**Duration bone pain**, months [IQR]	3 [2, 9]	**5 [2**,** 11]**	5 [2, 11]	4 [2, 11]	**3 [2**,** 6]**	3 [2, 5]	5 [2, 10]	0.292

**Fig. 2:**
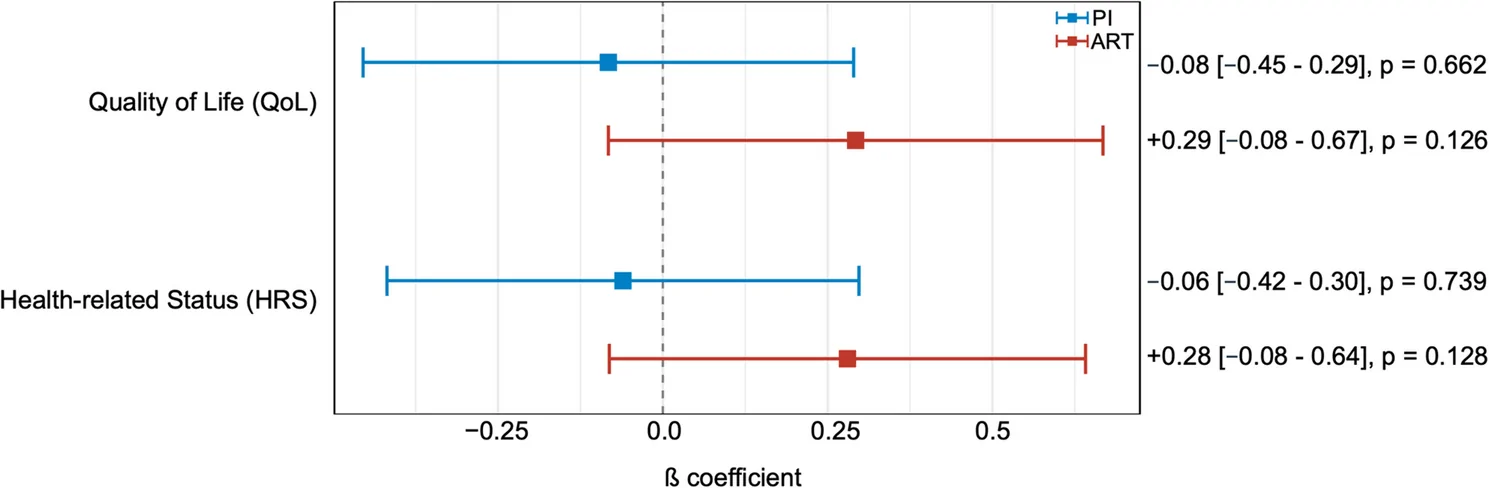
Adjusted associations of current proteasome inhibitor (PI) therapy and antiresorptive therapy (ART) with patient-reported quality of life (QoL) and health-related status. Forest plot showing β coefficients with 95% confidence intervals derived from multivariable linear regression models (*n* = 209). Models were adjusted for relevant clinical covariates and positive β coefficients indicate higher QoL or health-related status scores (scale 1–7). The vertical dashed line represents the null association (β = 0)

In multivariable linear regression, current PI-based treatment status was not associated with significantly altered QoL (β = -0.08, 95% CI -0.45 to 0.29; *p* = 0.662) or health-related status (β = -0.06, 95% CI -0.42 to 0.30; *p* = 0.740). ART was likewise not associated with significant changes in QoL (β = 0.29, 95% CI -0.08 to 0.67; *p* = 0.126) or health-related status (β = 0.28, 95% CI -0.08 to 0.64; *p* = 0.128; Fig. 2).

**Fig. 3:**
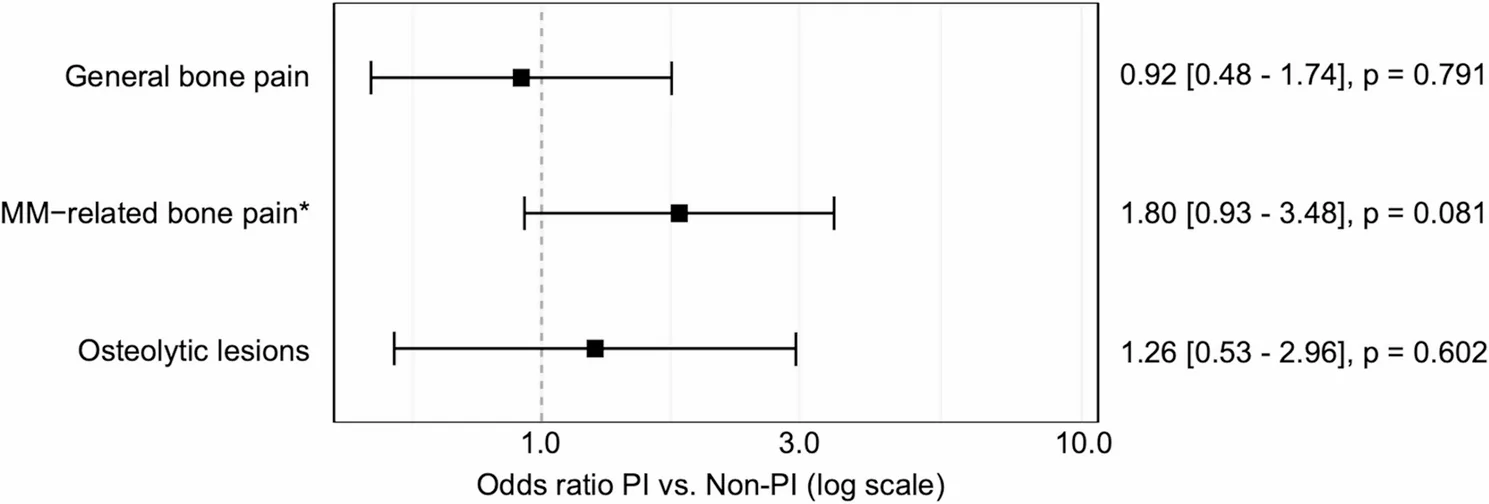
Adjusted associations between current proteasome inhibitor (PI) treatment status and binary clinical outcomes compared with non-PI treatment status. Forest plot showing odds ratios (OR) with 95% CI derived from multivariable logistic regression models. The vertical dashed line represents the null association (OR = 1) on the logarithmic scale. *MM-related bone pain was defined as bone pain attributed to MM, with patients without bone pain classified as not having MM-related bone pain (*n* = 209)

In multivariable logistic regression, current PI-based treatment status was not associated with prevalence of general bone pain (OR 0.92, 95% CI 0.48 to 1.74; *p* = 0.791) or osteolytic lesions (OR 1.26, 95% CI 0.53 to 2.96; *p* = 0.601). In the entire cohort, current PI-based treatment status was not significantly associated with MM-related bone pain (OR 1.80, 95% CI 0.93 to 3.48; *p* = 0.081; Fig. 3). Among patients reporting bone pain (*n* = 109), current PI-based treatment status was associated with higher odds of MM-related pain (OR 3.97, 95% CI 1.27 to 12.36; *p* = 0.017). In adjusted linear regression, current PI-based treatment status was not associated with differences in pain duration between treatment groups (β = 0.16 months, 95% CI -0.23 to 0.54; *p* = 0.425). Likewise, disease duration did not differ significantly between patients with PI-based and non-PI-based treatment status (β = -13.32 months, 95% CI -28.25 to 1.61; *p* = 0.080).

Additional exploratory analyses assessed potential interaction effects between PI and ART in combinational treatment regimens by including a PI × ART interaction term. No significant PI × ART interaction was observed for QoL (β = 0.41, 95% CI -0.32 to 1.15; *p* = 0.267) or health-related status (β = 0.21, 95% CI -0.51 to 0.92; *p* = 0.573). Further, no significant interaction was observed for general bone pain (OR 0.53, 95% CI 0.14 to 1.96; *p* = 0.343), MM-related pain (OR 0.51, 95% CI 0.14 to 1.84; *p* = 0.303), bone pain duration (β = 0.31, 95% CI -0.46 to 1.09; *p* = 0.424), or osteolytic lesions (OR 1.44, 95% CI 0.22 to 9.31; *p* = 0.704).

### Longitudinal changes in patient-reported outcomes and skeletal symptoms

The longitudinal cohort comprised 69 patients, of whom 27 (39%) were receiving PI-based therapy at baseline. Median follow-up was 12 months (IQR 8–16, range 6–24). During follow-up, PI status changed in 17 patients (24.6%; 10 discontinued and 7 initiated PI-based therapy), while ART status changed in 18 (26.1%; 5 discontinued and 13 initiated ART). Overall, 29 patients (42.0%) changed PI and/or ART status during follow-up. These changes resulted in small and imbalanced treatment subgroups at follow-up, particularly the PI+/ART− subgroup, which comprised only one patient. Accordingly, subgroup-based longitudinal comparisons should be interpreted cautiously. Baseline characteristics of the longitudinal cohort are presented in Table 3.

**Table 3:** Baseline characteristics of the longitudinal follow-up cohort (*n* = 69), stratified by proteasome inhibitor (PI) therapy at study inclusion. Continuous variables are presented as median [IQR] and categorical variables as counts n (%). *Percentage calculated within the full cohort (*n* = 69); patients without bone pain are classified as not having MM-related bone pain. Osteolytic-lesion denominators reflect non-missing imaging assessments. ART, antiresorptive therapy; CRAB, hypercalcemia/renal insufficiency/anemia/bone lesions; ISS, International Staging System; HRS, health-related status; QoL, quality of life

	Total (*n* = 69)	PI (*n* = 27)	Non-PI (*n* = 42)	*P* value
**Age**, median years [IQR]	69 [60, 74]	70 [63, 75]	68 [60, 73]	0.322
**Male sex**, n (%)	49 (71%)	19 (70%)	30 (71%)	1.000
**Antiresorptive therapy (ART)**, n (%)	44 (64%)	13 (48%)	31 (74%)	0.056
**CRAB symptoms**, median [IQR]				0.345
0	3 (4%)	0 (0.0%)	3 (7%)	
1	23 (33%)	11 (41%)	12 (29%)	
2	20 (29%)	7 (26%)	13 (31%)	
3	14 (20%)	4 (15%)	10 (24%)	
4	9 (13%)	5 (19%)	4 (10%)	
**ISS stage**, median [IQR]				0.666
I	22 (32%)	7 (26%)	15 (36%)	
II	20 (29%)	9 (33%)	11 (26%)	
III	27 (39%)	11 (41%)	16 (38%)	
**Orthopedic comorbidity**, n (%)	37 (54%)	16 (59%)	21 (50%)	0.613
**Bone pain**, n (%)	44 (64%)	18 (67%)	26 (62%)	0.885
MM-related bone pain*, n (%)	31 (45%)	15 (56%)	16 (38%)	0.240
**Duration bone pain**, months [IQR]	3 [2, 6]	4 [2, 6]	3 [1, 4]	0.057
**Osteolytic lesions**, n (%)	59 (88%)	25 (93%)	34 (85%)	0.578
**Progressive disease status**, n (%)	8 (12%)	5 (19%)	3 (7%)	0.247
**QoL score** (1–7), median [IQR]	5 [4, 6]	5 [4, 5.5]	5 [4, 6]	0.376
**HRS score** (1–7), median [IQR]	5 [4, 6]	4 [4, 6]	5 [4, 6]	0.574

At follow-up, neither patient-reported outcome measure changed significantly compared with baseline. Median QoL remained 5 (IQR 4–6; Δ 0 [0, 1], *p* = 0.778), and median health-related status remained 5 (IQR 4–6; Δ 0 [0, 1], *p* = 0.288). Likewise, no significant within-patient changes were observed for skeletal outcomes. Bone pain prevalence decreased from 63.8% to 53.6% (Δ − 10.1%, *p* = 0.167), and MM-related bone pain from 44.9% to 36.2% (Δ − 8.7%, *p* = 0.263). The prevalence of osteolytic lesions was stable (88.1% vs. 89.6%; Δ + 1.5%, *p* = 1.000), as was the proportion of patients with progressive disease status (11.6% vs. 13.0%; Δ + 1.4%, *p* = 1.000). Of the nine cases with progressive disease at follow-up, four were confirmed radiologically, two showed radiological progress, and three were identified based on clinical data. Subgroup analyses of bone-pain resolution showed no association with treatment regimen. Among the 44 patients reporting bone pain at baseline, pain resolved in 5 of 18 PI-treated patients (27.8%) and 8 of 26 non-PI-treated patients (30.8%; *p* = 1.000). Similarly, pain resolution occurred in 7 of 26 patients receiving antiresorptive therapy (26.9%) and 6 of 18 patients without antiresorptive therapy (33.3%; *p* = 0.742) (Table 4).

**Table 4:** Within-patient changes between baseline and follow-up in the longitudinal cohort. Continuous variables are presented as median [IQR] and categorical variables as counts n (%). Total (n) reflects patients with complete data at both time points for the respective outcome. Δ denotes the change in proportion (percentage points) for binary outcomes or the median within-patient change for ordinal scores. HRS, health-related status; MM, multiple myeloma; QoL, quality of life

	Total (*n*)	Baseline	Follow-Up	Diff. Δ	*P* value
**QoL score** (1–7), median [IQR]	69	5 [4, 6]	5 [4, 6]	0 [0, 1]	0.778
**HRS score** (1–7), median [IQR]	69	5 [4, 6]	5 [4, 6]	0 [0, 1]	0.288
**Bone pain**, n (%)	69	44 (63.8%)	37 (53.6%)	-10.1%	0.167
**MM-related bone pain**, n (%)	69	31 (44.9%)	25 (36.2%)	-8.7%	0.263
**Osteolytic lesions**, n (%)	67	59 (88.1%)	60 (89.6%)	+ 1.5%	1.000
**Progressive disease status**, n (%)	69	8 (11.6%)	9 (13.0%)	+ 1.4%	1.000
**Pain resolution by PI status**	PI 18 / Non-PI 26	—	PI 5 (27.8%) / Non-PI 8 (30.8%)	−3.0%	1.000
**Pain resolution by ART status**	ART 26 / Non-ART 18	—	ART 7 (26.9%) / Non-ART 6 (33.3%)	−6.4%	0.742

**Fig. 4:**
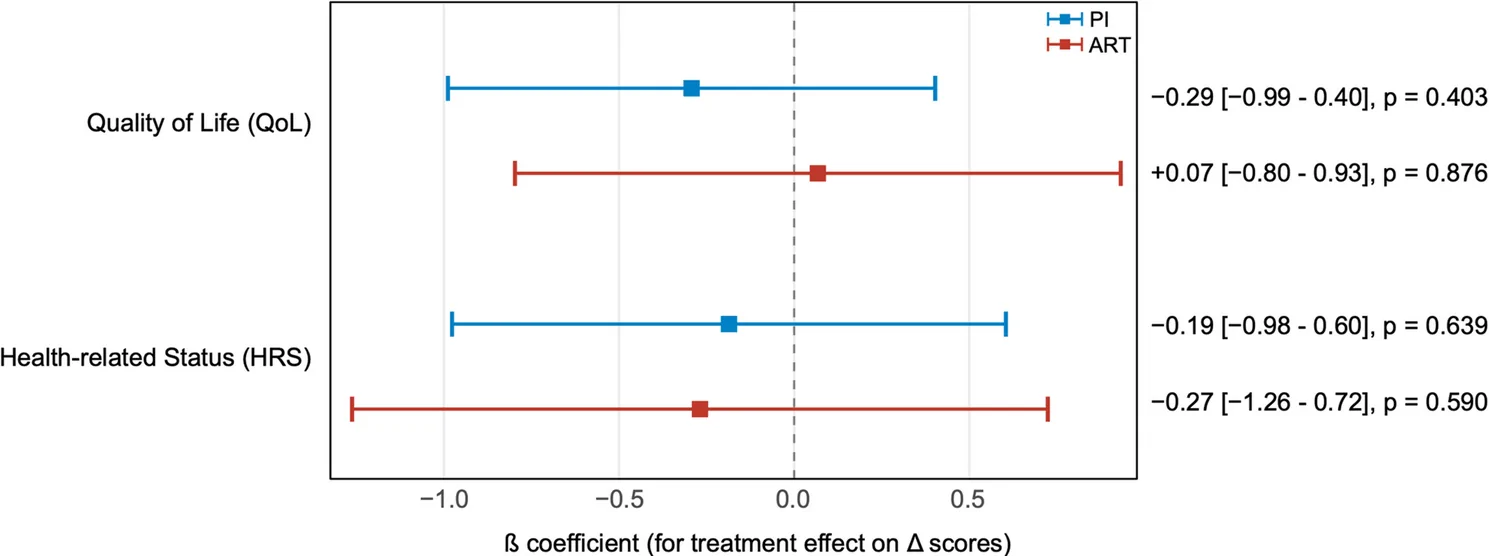
Longitudinal changes in PROMs by treatment regimen. Forest plot of adjusted linear regression analyses showing associations of current PI and ART status with change in quality of life (QoL) and health-related status between baseline and follow-up in the longitudinal follow-up cohort (*n* = 69). Points represent β coefficients with 95% confidence intervals. The vertical dashed line represents no change (β = 0)

Adjusted longitudinal linear regression of change scores (Fig. 4) showed no association of baseline PI-based treatment status with change in QoL (β = -0.29, *p* = 0.403) or HRS (β = -0.19, *p* = 0.639). Baseline ART was likewise not associated with change in QoL (β = 0.07, *p* = 0.876) or HRS (β = -0.27, *p* = 0.590). In adjusted longitudinal logistic regression, neither baseline PI nor baseline ART status was independently associated with prevalence of bone pain, MM-related bone pain, or osteolytic lesions at follow-up (Suppl. Figure 1).

In the exploratory interaction model (Suppl. Figure 2), a PI × ART interaction was observed for ΔQoL (β = -1.97, 95% CI -3.71 to -0.23; *p* = 0.027), whereas no significant interaction was observed for ΔHRS (β = -0.66, *p* = 0.545). Given the small and markedly imbalanced longitudinal subgroups, treatment switching during follow-up, and only one patient in the PI+/ART- subgroup at follow-up, this analysis should be considered exploratory and hypothesis-generating.

### Sensitivity analyses for baseline imbalance and confounding by indication

To assess the robustness of the principal findings, propensity score-based inverse-probability-of-treatment weighting (IPTW) was performed for the principal outcomes. The absence of an association between PI-based treatment status and patient-reported outcomes remained unchanged after weighting, with effect estimates close to the null for both QoL (β = -0.06, 95% CI -0.44 to 0.32; *p* = 0.752) and health-related status (β = -0.08, 95% CI -0.44 to 0.28; *p* = 0.663; Suppl. Figure 3A). Likewise, restricting the analyses to patients without progressive disease at study inclusion (*n* = 175) yielded comparable results for QoL (β = -0.02, 95% CI -0.40 to 0.36; *p* = 0.922) and health-related status (β = 0.05, 95% CI -0.32 to 0.42; *p* = 0.799).

In the entire cohort, the IPTW estimate for MM-related bone pain was OR 1.83 (95% CI 0.97 to 3.44; *p* = 0.061), consistent with the adjusted analysis. Among patients reporting bone pain, the association between current PI-based treatment status and MM-related bone pain remained significant after IPTW (OR 3.65, 95% CI 1.21 to 11.04; *p* = 0.022). Associations with general bone pain (OR 1.09, 95% CI 0.59 to 2.00; *p* = 0.790), osteolytic lesions (OR 1.46, 95% CI 0.65 to 3.30; *p* = 0.364), and pain duration (β = 0.19, 95% CI -0.22 to 0.59; *p* = 0.363) remained non-significant, consistent with the primary adjusted analyses (Suppl. Figure 3B). Restricting the multivariable analysis to patients without progressive disease at study inclusion (*n* = 175) yielded a comparable estimate among patients reporting bone pain (OR 4.19, 95% CI 1.15 to 15.25; *p* = 0.029) and an attenuated, non-significant estimate in the entire cohort (OR 1.62, 95% CI 0.77 to 3.41; *p* = 0.202). Full covariate-balance diagnostics for the IPTW model, including standardized mean differences before and after weighting, the propensity-score distribution by treatment group, and the stabilized weight distribution and effective sample size, are provided in Suppl. Table 4 and Suppl. Figure 4.

## Discussion

In this prospective interdisciplinary real-world cohort of 209 patients with multiple myeloma, current PI-based treatment status was not associated with measurable differences in global patient-reported QoL or health-related status, general bone pain, pain duration, or prevalent osteolytic lesions compared with non-PI-based treatment. The only outcome associated with current PI-based treatment status was MM-related bone pain, which was numerically more frequent in the entire cohort and showed a stronger association among patients reporting bone pain. These findings describe associations according to treatment status at study assessment and should not be interpreted as estimates of a causal PI treatment effect.

The association with MM-related bone pain requires caution because PI-based treatment regimens are preferentially used in patients with active, symptomatic, or progressive disease and may therefore be more common among patients with greater disease activity and skeletal symptom burden [20–22]. Consistent with differences in treatment history and disease phase, ASCT as part of primary therapy was less frequent among patients currently receiving PI-based treatment (58% vs. 79%). Similarly, ISS was the only standardized disease-stage measure consistently available across the cohort. More detailed risk-stratification systems such as R-ISS and mSMART could not be applied because the required cytogenetic and laboratory information was not consistently available. Thus, adjustment may not have captured the full clinical and biological heterogeneity underlying treatment selection.

In the entire cohort, MM-related bone pain was numerically more frequent among patients currently receiving PI-based treatment, but the association was attenuated and did not reach statistical significance after multivariable adjustment or IPTW. The association was stronger in the secondary analysis restricted to patients reporting bone pain, in which it persisted after multivariable adjustment and IPTW. After excluding patients with progressive disease at study inclusion, the association remained in the conditional analysis, whereas the estimate in the entire cohort was attenuated and remained non-significant. Thus, the association observed among patients reporting bone pain was not solely driven by those classified as having progressive disease. Nevertheless, residual confounding by treatment response, depth of remission, treatment line, treatment duration, previous treatment exposure, and physician-driven regimen selection cannot be excluded. Notably, overall bone-pain prevalence was comparable between groups, whereas a greater proportion of pain was attributed to MM among patients currently receiving PI-based treatment. Because attribution relied on concordance between pain location and osteolytic involvement on imaging, this pattern may reflect differences in underlying disease activity, skeletal disease distribution, or pain classification. These findings should therefore be regarded as descriptive associations and not as evidence of an independent pharmacological effect of current PI-based treatment.

By contrast, QoL and health-related status remained comparable between treatment groups across baseline, longitudinal, and sensitivity analyses. Neither baseline PI-based nor antiresorptive treatment status was associated with longitudinal changes in these patient-reported measures. However, treatment exposure was not stable during follow-up: a substantial proportion of patients changed PI and/or ART status, and the longitudinal models were based on baseline treatment classification. These analyses therefore reflect associations with treatment status at baseline rather than sustained exposure throughout follow-up. The exploratory PI × ART interaction for change in QoL should likewise be interpreted cautiously because of the small and markedly imbalanced longitudinal subgroups and requires confirmation in larger cohorts.

The absence of measurable differences in global patient-reported outcomes is broadly compatible with the heterogeneous literature on PI-containing regimens. In most clinical trials, PROMs have been assessed as secondary endpoints of combination regimens rather than as treatment-specific effects and have varied considerably regarding methodology, instruments, and reporting [23, 24]. Trials evaluating Bortezomib- and carfilzomib-based regimens, including ASPIRE [25], APEX [26], and ENDEAVOR [27], generally demonstrated maintained or modestly improved QoL, although effects were inconsistent across domains and often below thresholds for clinical relevance, particularly regarding pain. Likewise, the TOURMALINE trials [28–30] reported largely preserved patient-reported outcomes despite improvements in progression-free survival. These data therefore provide limited evidence regarding a treatment-specific effect of proteasome inhibition on patient-reported skeletal outcomes.

This distinction is particularly relevant to skeletal disease. Preclinical and translational studies have demonstrated favorable effects of proteasome inhibition on osteoclastogenesis, osteoblast differentiation, osteocyte viability, and bone-remodeling biomarkers [13, 15, 17, 19]. However, evidence that these biological effects translate into reductions in incident skeletal lesions, skeletal-related events, or bone pain independently of systemic disease control remains limited and difficult to differentiate from concomitant bone-targeted therapy [14, 31, 32]. Moreover, prevalent osteolytic lesions primarily represent cumulative structural skeletal damage rather than contemporaneous bone-remodeling activity. Established lytic defects may persist despite effective antimyeloma treatment and deep hematologic response, while radiographic reconstitution may be incomplete or require prolonged follow-up [33, 34]. The binary presence of osteolytic lesions in the present study should therefore not be interpreted as a sensitive surrogate for short-term treatment-associated changes in bone remodeling.

More broadly, skeletal symptoms in MM are inherently heterogeneous and multifactorial, reflecting not only current disease activity but also persistent osteolytic damage, previous skeletal complications, concomitant musculoskeletal disorders, analgesic treatment, and individual pain perception [11, 35–37]. Within this complexity, the present study provides a clinically relevant real-world perspective by integrating treatment status with patient-reported symptoms and radiological skeletal findings during routine interdisciplinary follow-up. Notably, global QoL and health-related status were comparable between patients receiving current PI-based and non-PI-based treatment. At the same time, the distinction between overall and MM-related bone pain highlights the clinical importance of considering the underlying cause of pain when assessing skeletal symptoms in patients with MM. These findings provide clinically informative descriptive data but do not establish whether the biological effects of proteasome inhibition translate into patient-reported or skeletal benefits beyond its systemic antimyeloma efficacy [38, 39].

### Strengths and limitations

Strengths of this study include its prospective real-world design and interdisciplinary assessment integrating hematological treatment information with orthopedic, radiological, and patient-reported outcomes during routine clinical follow-up. This approach enabled complementary assessment of overall bone pain, its attribution to MM-related skeletal disease, structural skeletal involvement, and global patient-reported status. Longitudinal follow-up and complementary multivariable and propensity-score sensitivity analyses further strengthened the descriptive characterization of these outcomes according to current treatment status.

Several limitations should nevertheless be considered. Most importantly, treatment allocation was non-random, and residual confounding cannot be eliminated despite adjustment and IPTW. Analgesic and opioid use was not systematically captured and could therefore not be incorporated into analyses of pain-related outcomes.

In addition, QoL and health-related status were assessed using pragmatic single-item 1-7-Likert ratings rather than validated multidomain instruments such as the EORTC QLQ-C30, QLQ-MY20, or EQ-5D [23]. Although these global ratings facilitate brief assessment during routine clinical follow-up, they provide limited information on specific domains such as physical functioning, fatigue, symptoms, or treatment-related toxicity and may therefore be less sensitive to subtle treatment-associated changes. The longitudinal cohort was relatively small, treatment status frequently changed during follow-up, and the observation period may have been insufficient to detect delayed structural skeletal changes, as bone remodeling can evolve over substantially longer periods. Further, the binary osteolysis endpoint did not distinguish stable lesions from radiographic healing or sclerosis, although radiological progression was assessed separately within the progressive-disease evaluation. Finally, the single-center setting within a tertiary interdisciplinary myeloma program may limit the generalizability of the findings to other institutions, treatment settings, and patient populations.

Future studies should incorporate standardized hematologic response assessment (e.g., International Myeloma Working Group (IMWG) response criteria [40]), time-varying treatment exposure, longitudinal skeletal imaging, validated multidomain PROMs, and detailed characterization of skeletal disease burden to determine whether the biological effects of proteasome inhibition translate into clinically meaningful patient benefit.

## Conclusions

In this prospective interdisciplinary real-world cohort, current PI-based treatment status was not associated with measurable differences in global patient-reported QoL and health-related status, general bone pain, pain duration, or prevalent osteolytic lesions compared with non-PI-based treatment. MM-related bone pain was numerically more frequent among patients currently receiving PI-based treatment, although the association did not reach statistical significance after adjustment and residual confounding by disease and treatment characteristics cannot be excluded. Together, these findings provide a clinically informative real-world description of patient-reported and skeletal outcomes according to current treatment status and underscore the importance of considering pain etiology when assessing skeletal symptoms in patients with MM. However, they do not establish whether the biological effects of proteasome inhibition on bone remodeling translate into clinically meaningful skeletal or patient-reported benefits.

## Electronic Supplementary Material

Below is the link to the electronic supplementary material.

Supplementary Material

## Data Availability

The pseudonymized datasets analyzed during the current study contain potentially identifiable patient information and are therefore not publicly deposited, in accordance with the institutional data-protection policy and the consent obtained from participants. They are available from the corresponding author upon reasonable request and subject to a data-sharing agreement and approval by the local ethics committee. The R script that reproduces all analyses reported in this paper is available from the corresponding author upon reasonable request.
