# Supplementary material for: Patient-reported outcomes and skeletal symptoms in multiple myeloma stratified by proteasome inhibitor-based therapy: a prospective interdisciplinary cohort study
Source: BMC Cancer. 2026 Oct 15;26:1290. doi: 10.1186/s12885-026-17177-7 (PMC13695283; doi:10.1186/s12885-026-17177-7)
Supplement: Supplementary file 1 [file 12885_2026_17177_MOESM1_ESM.docx]

**Supplementary**

**Suppl. Fig. 1.** Longitudinal logistic regression in the follow-up cohort (n = 69). Adjusted odds ratios (OR) with 95% CI for associations of baseline proteasome-inhibitor (PI) therapy and antiresorptive therapy (ART) with binary follow-up outcomes. The vertical dashed line indicates the null association (OR = 1). *MM-related bone pain was analyzed in the entire follow-up cohort (n = 69); patients without bone pain were classified as not having MM-related bone pain.

**Suppl. Fig. 2.** Forest plot of adjusted longitudinal linear regression analyses showing associations of PI, ART, and their interaction (PI × ART) with change in QoL and health-related status between baseline and follow-up in the longitudinal follow-up cohort (n = 69). Points represent β coefficients and horizontal lines indicate 95% CI. The vertical dashed line indicates the null association (β = 0).

**Suppl. Fig. 3.** Sensitivity analysis comparing the estimated association of proteasome-inhibitor (PI) therapy with each principal outcome under two approaches: multivariable adjusted regression (Adjusted) and inverse-probability-of-treatment weighting (IPTW). (A) Shows β coefficients with 95% confidence intervals for continuous outcomes. (B) Shows odds ratios (OR) with 95% confidence intervals on the log scale for binary outcomes. *MM-related bone pain was defined as bone pain attributed to MM, with patients without bone pain classified as not having MM-related bone pain (n = 209). The vertical dashed line indicates the null association (A: β = 0; B: OR = 1).

**Suppl. Fig. 4.** Propensity-score weighting diagnostics. (A) Love plot showing absolute standardized mean differences (SMDs) for the nine propensity-score covariates before and after stabilized inverse probability of treatment weighting (IPTW). The dashed vertical line indicates the prespecified balance threshold of |SMD| = 0.10. (B) Distribution of estimated propensity scores according to current PI exposure, illustrating overlap between PI-exposed and non-PI patients. (C) Distribution of stabilized average treatment effect (ATE) weights after truncation at the 1st and 99th percentiles. After weighting, all covariates achieved |SMD| < 0.10.

| Concomitant therapy / Agent | Total (n=209) | PI (n=78) | Non-PI (n=131) | P value |
| --- | --- | --- | --- | --- |
| Immunomodulatory drug (IMiD) | **84 (40%)** | **5 (6%)** | **79 (60%)** | **<0.001** |
| *Lenalidomide* | 69 (33%) | 5 (6%) | 64 (49%) | <0.001 |
| *Pomalidomide* | 15 (7%) | 0 (0%) | 15 (11%) | 0.005 |
| Immunotherapy (monoclonal/bispecific antibody) | **80 (38%)** | **24 (31%)** | **56 (43%)** | **0.115** |
| *Daratumumab* | 63 (30%) | 22 (28%) | 41 (31%) | 0.752 |
| *Isatuximab* | 2 (1%) | 2 (3%) | 0 (0%) | 0.138 |
| *Elotuzumab* | 6 (3%) | 0 (0%) | 6 (5%) | 0.086 |
| *Teclistamab* | 5 (2%) | 0 (0%) | 5 (4%) | 0.160 |
| *Talquetamab* | 3 (1%) | 0 (0%) | 3 (2%) | 0.295 |
| *Belantamab* | 2 (1%) | 0 (0%) | 2 (2%) | 0.530 |
| CAR-T cell therapy | **4 (2%)** | **0 (0%)** | **4 (3%)** | **0.299** |
| Antiresorptive therapy (ART) | **111 (53%)** | **38 (49%)** | **73 (56%)** | **0.402** |
| *Bisphosphonate* | 85 (41%) | 28 (36%) | 57 (44%) | 0.348 |
| *Denosumab* | 26 (12%) | 10 (13%) | 16 (12%) | 1.000 |

**Suppl. Tab. 1.** Concomitant therapies at study inclusion according to current PI treatment status. Distribution of immunomodulatory drugs (IMiDs), immunotherapies, CAR-T cell therapy, and antiresorptive therapy (ART), including individual agents, among the total cohort and stratified by current PI-based versus non-PI-based treatment. Data are presented as n (%). ART comprised bisphosphonates and denosumab.

| Agent | Baseline (n=78) | Follow-up (n=24) |
| --- | --- | --- |
| *Bortezomib* | 62 (79%) | 17 (71%) |
| *Carfilzomib* | 11 (14%) | 4 (17%) |
| *Ixazomib* | 5 (6%) | 3 (13%) |

**Suppl. Tab. 2.** Distribution of individual proteasome inhibitors among PI-exposed patients at baseline and follow-up. Number and proportion of PI-exposed patients receiving bortezomib, carfilzomib, or ixazomib at baseline and follow-up. Percentages refer to the respective PI-exposed cohort at each assessment.

| Treatment combination | n | % |
| --- | --- | --- |
| PI-exposed (n = 78) | | |
| *Bortezomib* | 25 | 32.1% |
| *Bortezomib + Bisphosphonate* | 14 | 17.9% |
| *Bortezomib + Daratumumab* | 9 | 11.5% |
| *Bortezomib + Denosumab* | 6 | 7.7% |
| *Bortezomib + Daratumumab + Bisphosphonate* | 4 | 5.1% |
| *Carfilzomib + Bisphosphonate* | 3 | 3.8% |
| *Carfilzomib + Daratumumab* | 2 | 2.6% |
| *Carfilzomib + Daratumumab + Bisphosphonate* | 2 | 2.6% |
| Other/unique combinations (13 distinct, 1 patient each) | 13 | 16.7% |
| Non-PI (n = 131) | | |
| *Lenalidomide + Bisphosphonate* | 33 | 25.2% |
| *ASCT* | 25 | 19.1% |
| *Bendamustine + Dexamethasone* | 1 | 0.8% |
| *Lenalidomide + Daratumumab* | 8 | 6.1% |
| *Pomalidomide + Daratumumab + Bisphosphonate* | 8 | 6.1% |
| *Daratumumab* | 7 | 5.3% |
| *Lenalidomide + Denosumab* | 7 | 5.3% |
| *Lenalidomide + Daratumumab + Bisphosphonate* | 6 | 4.6% |
| *Lenalidomide* | 5 | 3.8% |
| *Pomalidomide + Elotuzumab* | 4 | 3.1% |
| *ASCT + Bisphosphonate* | 3 | 2.3% |
| *CAR-T* | 3 | *2.3%* |
| *Lenalidomide + Daratumumab + Denosumab* | 3 | 2.3% |
| *Daratumumab + Denosumab* | 3 | 2.3% |
| *Daratumumab + Teclistamab* | 2 | 1.5% |
| *Lenalidomide + Elotuzumab + Bisphosphonate* | 2 | 1.5% |
| *Teclistamab + Bisphosphonate* | 2 | 1.5% |
| Other/unique combinations (9 distinct, 1 patient each) | 9 | 6.9% |

**Suppl. Tab. 3.** Treatment combinations at study inclusion according to current PI status (n = 209).
Combinations occurring in only one patient within a treatment group were pooled as “Other/unique combinations”. Bisphosphonates and denosumab are supportive antiresorptive therapies rather than antimyeloma agents but are included because of their relevance to skeletal outcomes. ASCT denotes autologous stem cell transplantation as the most recent myeloma-directed treatment at the study assessment. Percentages refer to the respective PI-exposed or non-PI subgroup.

| Covariate | SMD, unweighted | SMD, IPTW-weighted |
| --- | --- | --- |
| Orthopedic comorbidity | +0.034 | +0.018 |
| Antiresorptive therapy (ART) | -0.070 | +0.002 |
| Progressive disease | +0.027 | -0.009 |
| Male sex | -0.087 | +0.016 |
| Age | +0.180 | +0.075 |
| ASCT | -0.209 | -0.001 |
| CRAB score | +0.220 | -0.004 |
| ISS stage | +0.304 | +0.043 |
| Disease duration | -0.346 | +0.030 |

**Suppl. Tab. 4.** **Covariate balance before and after inverse probability of treatment weighting (IPTW).**
Standardized mean differences (SMDs) are shown for the nine propensity-score covariates before weighting and after stabilized average treatment effect weighting with truncation at the 1st and 99th percentiles. For binary covariates, SMDs represent differences in proportions. Continuous covariates were standardized using the pooled standard deviation. Signed values indicate the direction of the difference (PI-exposed minus non-PI). The propensity-score analysis included all 209 patients. Effective sample sizes after weighting were 64.2 for PI-exposed patients (n = 78) and 121.0 for non-PI patients (n = 131). Truncated weights had a median of 0.90 (range 0.55-2.45). All covariates achieved |SMD| < 0.10 after weighting, consistent with adequate covariate balance. Absolute SMDs and corresponding propensity-score and weight-distribution diagnostics are shown in **Suppl. Fig. 4.**
